# Communication Strategies Used to Obtain Clinical Histories Before Remotely Prescribing Antibiotics for Postal Treatment of Uncomplicated Genital Chlamydia: Service Evaluation

**DOI:** 10.2196/15970

**Published:** 2020-06-17

**Authors:** Hannah McCulloch, Jonathan Syred, Gillian Holdsworth, Chris Howroyd, Elena Ardines, Paula Baraitser

**Affiliations:** 1 School of Population Health & Environmental Sciences King's College London London United Kingdom; 2 SH:24 London United Kingdom; 3 Department of Sexual Health and HIV King's College Hospital London United Kingdom

**Keywords:** sexual health, electronic prescribing, telemedicine, remote consultation, sexually transmitted diseases, bacterial

## Abstract

**Background:**

Web-based services for testing of sexually transmitted infections are widely available across the United Kingdom. Remote prescriptions with medications posted home may support prompt treatment; however, the absence of face-to-face contact with clinicians raises clinical safety issues as medical history may not be accurately provided.

**Objective:**

This service evaluation aimed to capture the use and explore the safety of 3 remote communication strategies employed within a web-based service offering remote prescriptions of antibiotics, delivered via post, for uncomplicated genital *Chlamydia trachomatis*. User acceptability and time-from-diagnosis-to-treatment were also obtained.

**Methods:**

Three iterations of the service were compared, where medical history was collected via SMS text message, telephone, or a secure web form before a prescription was issued. We contacted users after they were issued a prescription and completed the medical history a second time via telephone, asking when they took their medication and how they felt about the service. The primary safety measure was agreement in information supplied at 2 assessments (ie, clinical and evaluation assessment) on key elements of safe prescribing: allergies, current medications, or contraindicating clinical conditions or symptoms. Agreement in information between clinical and evaluation assessment was summarized as a binary variable. Factors associated with the assessment agreement variable were explored using univariate and multivariate analysis. The secondary evaluation measures were recall of and adherence to instructions for taking medication, time-from-diagnosis-to-treatment, and acceptability of the web-based service.

**Results:**

All web-based service users, resident in the London Boroughs of Lambeth and Southwark with a positive chlamydia diagnosis, who were eligible for and chose postal treatment between February 15, 2017, and October 24, 2017, were invited to participate in this service evaluation. Of 321 eligible users, 62.0% (199) participated. A total of 27.6% (55/199) users completed the clinical assessment via SMS text message, 40.7% (81/199) users via telephone, and 31.7% (63/199) users via a secure web form. Those who were assessed for prescription via SMS text message were less likely to have an agreement in safe prescribing information than those assessed via telephone (adjusted odds ratio [aOR] 0.22, 95% CI 0.08-0.61; *P*=.004). We found no statistically significant difference in odds of agreement between the web form and telephone assessment (aOR 0.50, 95% CI 0.17-1.43; *P*=.20). Median time-to-treatment was 4 days (IQR 3-5.5). In addition, 99.0% (196/199) of users reported understanding remote communication, and 89.9% (178/198) would use the service again.

**Conclusions:**

Postal treatment is an acceptable and rapid treatment option for uncomplicated genital chlamydia. Clinical assessment via SMS text message before remote prescription may not be accurate or sufficient. As health care is delivered via the web, strategies that support safe remote prescribing are increasingly important, as is their evaluation, which should be robust and carefully considered.

## Introduction

### Background

Web-based services for testing for sexually transmitted infections (STI) with test kits sent home are widely implemented in the United Kingdom [1,2]. In these services, users order a test kit from a web-based service, take the samples themselves, and post them to a laboratory with results given via SMS text message or telephone. There is evidence that they increase testing uptake in comparison with clinic-based services [3], but the clinical and public health impact of better access to testing requires prompt treatment of the diagnosed infections to prevent onward transmission. Remote prescriptions with medication posted home may facilitate prompt treatment.

Remote prescriptions without face-to-face health professional contact raise safety concerns [4]. The UK General Medical Council advises that clinicians should be satisfied in their ability to adequately assess the patient’s condition through satisfactory medical and drug history, including allergies before remote prescribing, and recommends consideration of the limitations of the medium of communication used to ascertain this information [5]. Health Care Improvement Scotland and the UK National Prescribing Centre have made similar recommendations [4,6,7], advocating a cautious approach to remote prescribing. This is particularly important when prescribing treatment for chlamydia within a web-based sexual health service because (1) there is no face-to-face contact throughout the process of testing, diagnosis, and treatment; (2) sexual health service records are not linked to the primary care record making it difficult to cross-check information, and (3) instructions for use and partner notification are provided remotely.

In this context, it is therefore, particularly important that there is effective remote communication to obtain accurate and sufficient medical history for prescribing, and to ensure correct use of medication and partner notification.

Research has documented poor practice in remote prescribing with medications available without a prescription or an appropriate medical history [8,9]; there are obvious health risks associated with this poor practice [10,11]. However, remote prescribing is also delivered by highly regulated providers where doctors obtain clinical histories through telephone, video, or email consultations [12,13]. There is good evidence that self-completed digital forms collect reliable data on past medical histories for diagnosis [14-17], but little evidence on their value before prescribing. As many health systems move toward a *digital first* approach (see, eg, the National Health Service, NHS long-term plan [18]) and increasingly use digital or web-based forms for clinical histories, we predict a need to understand their ability to generate accurate information for prescribing.

We developed and piloted remote prescribing and postal treatment of uncomplicated chlamydia infection within a web-based sexual health service. The process and outcomes of testing have been described elsewhere [3,19]. This service was evaluated during its implementation and development to monitor its clinical safety. As the service evolved, 3 remote communication strategies were used to take clinical histories before the prescribing and postal delivery of treatment of uncomplicated chlamydia. As the service evolved, the accuracy of the information obtained through each remote communication strategy was checked by telephoning users after they had received their prescriptions to recheck the information that they had provided. We sought to understand the safety issues highlighted by this service improvement activity. To do this, we described 3 cycles of service development to understand:

- How different media of assessment support accurate medical histories for prescribing
- To what extent service users understood remotely delivered instructions for use
- How different strategies for remote prescription and treatment impacted on time-to-treatment

### The Service Evaluated

Sexual Health 24 hours a day (SH:24) is an NHS commissioned web-based service that specializes in system transformation in sexual health services through agile and design-led thinking, using an iterative process of build/test/learn for innovation. SH:24 offers postal self-sampling test kits for chlamydia, gonorrhea, HIV, and syphilis. Test kits are ordered through a website, with those reporting symptoms of infection referred to local clinics. Test kits are sent by post in discreet packaging that includes the materials for self-sampling—urine for men and vaginal samples for women—and a finger-prick blood sample for HIV and syphilis. The user posts the samples to a laboratory for testing. Chlamydia, gonorrhea, syphilis, and negative HIV test results are delivered via SMS text message. Reactive HIV test results are delivered via telephone with referral to local clinics for confirmatory testing.

Those with positive chlamydia results who are older than 18 years and asymptomatic are offered postal chlamydia treatment and complete a remote assessment to check their eligibility for the appropriate antibiotic. If eligible, an electronic prescription is issued by a clinician, and the medication is dispensed, packaged, and dispatched by a regulated pharmacy through the Royal Mail registered (but not signed for) postal service. If ineligible for the treatment offered by the web-based service, which at the time of evaluation was a single 1 g dose of Azithromycin (SDA) users would be directed to local clinic services for treatment.

During and in response to the evaluation, the media of communication for assessment before the remote prescription changed 3 times.

1. During the first iteration of the service, eligibility was assessed via SMS text message. A single message rather than multiple messages was used as a break in communication during a process (eg, a loss of phone connection) would disrupt the assessment process. The message stated the following:

Your chlamydia result is positive. We would like to post you a single dose treatment. You would receive it the next working day. First, we need to know: Are you taking medicines or allergic to any medicines, soya or peanuts? (this is important because the medicine may trigger your allergy or interact with other medicines). Do you have liver, kidney, heart problems, or myasthenia gravis? (this is important because treatment may worsen your condition). Are you pregnant or breastfeeding? (this is important as the treatment may affect your baby). Do you have any symptoms: fever, joint pain, pelvic (lower abdominal) pain, or anal pain? (this is important as you may need a different treatment). If you answered no to ALL of these questions, reply NO. If you answered yes to ANY of these questions, reply YES. If you would like to go to a clinic for treatment instead, please reply CLINIC. Text back if you would like help. Thanks, SH:24.

Those that responded no were then sent this message.

Thank you. Your treatment is now being prepared. You have told us that you are not taking any medicines, you do not have any medical problems (liver, kidney, heart, myasthenia gravis) and you do not have any symptoms of chlamydia. Text back if you have any queries or questions⸺we always prefer to answer queries to prevent any future problems. Thanks, SH:24

2. During the second iteration, the same questions were asked by a clinician over the telephone with responses recorded in the web-based clinical record.

3. During the third iteration, the same questions were asked via a secure web form, which was self-completed by service users.

Instructions for medication use, including abstinence from sexual intercourse for 7 days after commencing treatment and partner notification was provided in written form with the medication. The change from iteration 1 to iteration 2 occurred because a user reported an allergy in the evaluation that was not reported in the clinical assessment. The change from iteration 2 to iteration 3 occurred because it was felt to be time inefficient for the clinical team. Iteration 3 is the system currently implemented.

## Methods

### Design and Data Collection

All SH:24 users, resident in the London Boroughs of Lambeth and Southwark, with a positive chlamydia diagnosis who were eligible for and chose postal treatment between February 15, 2017, and October 24, 2017, were invited to take part in this service evaluation. Data were collected through a standardized telephone questionnaire delivered by a trained researcher after the medication had been prescribed and dispatched. This questionnaire documented relevant medical history, medication history (including allergies), time-to-treatment, and user experience. The full questionnaire can be found in Multimedia Appendix 1.

**Figure 1:**
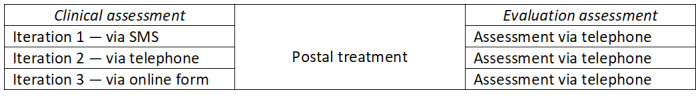
Data collection points and methods.

Each user, in effect, received the medical eligibility questions twice—once during their clinical assessment and once during the service evaluation. In iterations 1 and 3, there was a difference in the mode of communication between the clinical assessment and the evaluation assessment. During iteration 2, the mode of questioning before and after treatment delivery was the same. This is illustrated in Figure 1. As this was a service evaluation, there were no power calculations to determine the number of people in each arm. The service developed in response to real-time analysis of the evaluation rather than according to a research plan.

### Measures

We refer to the preprescription assessment as the clinical assessment and the postprescription assessment as the evaluation assessment. The primary safety measure was agreement in information supplied at the 2 assessments on key elements of safe prescribing: allergies, current medications, or contraindicating clinical conditions or symptoms. The secondary evaluation measures were recall of and adherence to instructions for taking the medication, time-from-diagnosis-to-treatment, and acceptability of the postal treatment service.

### Analysis

Descriptive statistics were used to summarize participant characteristics using parametric and, where appropriate, nonparametric tests. Agreement in key safe prescribing information supplied at the clinical assessment and the evaluation assessment was summarized as 1 binary variable, where matching information between assessments was given a value of 1, and conflicting or disparate information was given a value of 0. Logistic regression was then used to determine factors associated with agreement in key safe prescribing information. Univariate analysis was used to determine its relationship with demographic characteristics, previous clinic attendance, and method of clinical assessment, and variables significant at a 0.05 level were compiled to create a multivariable logistic model. A Kruskal-Wallis H test was conducted to determine the relationship between median time-to-treatment and method of clinical assessment.

### Ethical Considerations

This work observed a build-test-learn cycle based on a human-centred design approach to service development carried out by a digital sexual health service, which adapted strategies that are already used by UK-registered web-based pharmacies. As a service evaluation, it focuses on auditable outcomes such as compliance with medical advice and processes, as well as time to treatment. For this service evaluation project, we did not request a formal ethics review; however, we conducted the evaluation in line with standard ethical procedures. To maintain confidentiality, users were asked to confirm the first line of their address before any information was disclosed about the nature of the telephone call. They were given full information about the evaluation and given the option to participate. Paper surveys, filled in by the researcher while on the phone, were marked with the user’s unique identifier and stored in a locked filing cabinet. Unique identifiers could only be linked to identifiable data by accessing the web-based service.

## Results

### Evaluation Uptake

**Figure 2:**
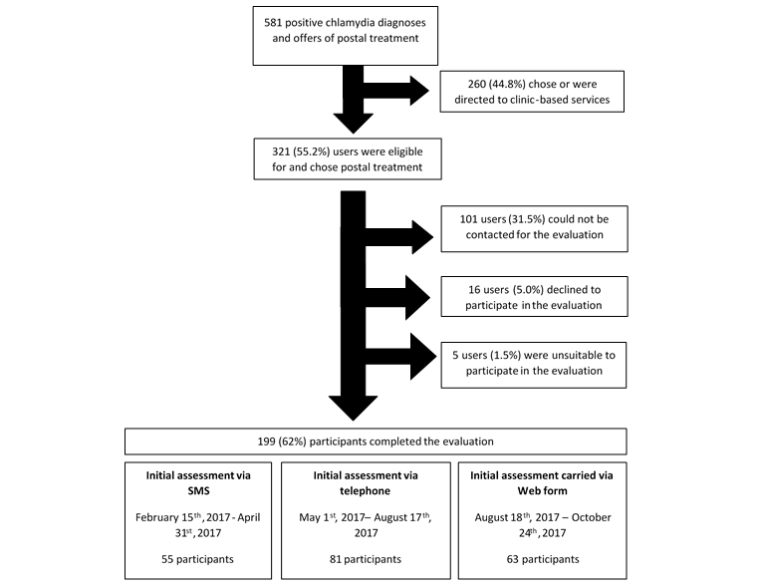
Evaluation flowchart.

During the study period, there were 581 chlamydia diagnoses and offers of postal treatment, of which 260 (44.8%) chose to access or were directed to clinic-based services (mainly because they had symptoms). Of the 321 who chose postal treatment, 199 (62.0%) participated in the evaluation (101 could not be contacted, 16 declined to participate, and 5 were excluded—3 repeat users, 1 could not confirm their address, and 1 had decided to attend a clinic). Of the 199 participants, 55 (27.6%) completed the clinical assessment via SMS text message, 81 (40.7%) via telephone, and 63 (31.7%) via a secure web form (Figure 2). The median time between the clinical assessment and the evaluation assessment was 17 days (IQR 10-35).

### Participant Characteristics

The sociodemographic and clinic attendance characteristics of those who did and did not take part in the evaluation are described in Table 1.

**Table 1:** Sociodemographic characteristics of individuals who ordered postal treatment for chlamydia (N=321).

Sociodemographic characteristic		Sample (n=199), n (%)	Population (N=321), n (%)
**Age group (years)**			
	18-19	21 (10.6)	37 (11.5)
	20-24	83 (41.7)	126 (39.3)
	25-29	54 (27.1)	95 (29.6)
	30-34	26 (13.1)	39 (12.1)
	35+	15 (7.5)	24 (7.5)
**Gender**			
	Male	99 (49.7)	168 (52.3)
	Female	100 (50.3)	153 (47.7)
**Ethnicity**			
	White	76 (38.2)	143 (44.6)
	Black British or Black Caribbean or Black African	73 (36.7)	103 (32.1)
	Asian or Asian British	8 (4.0)	12 (3.7)
	Mixed or multiple groups	24 (12.1)	38 (11.8)
	Other or prefer not to say	18 (9.0)	25 (7.8)
**Sexual orientation**			
	Heterosexual	176 (88.5)	288 (89.7)
	Homosexual	5 (2.5)	7 (2.2)
	Bisexual	14 (7.0)	17 (5.3)
	Prefer not to say	4 (2.0)	9. (2.8)
**Index of multiple deprivation** **quintile**			
	1 (most deprived)	66 (33.2)	108 (33.6)
	2	83 (41.7)	127 (39.6)
	3	32 (16.1)	56 (17.5)
	4	13 (6.5)	19 (5.9)
	5 (least deprived)	5 (2.5)	11 (3.4)
**Ever attended a sexual health clinic?**			
	Yes	147 (73.9)	227 (70.7)
	No	52 (26.1)	94 (29.3)
**Attended a sexual health clinic in the last 12 months?^a^**			
	Yes	77 (38.7)	109 (34.0)
	No	94 (47.2)	150 (46.7)
	Did not answer	28 (14.1)	62 (19.3)

^a^Was not answered by all participants.

### Agreement in Key Safe Prescribing Information

We considered all discrepancies between the clinical and evaluation assessment. New clinical information reported at the evaluation assessment but not the clinical assessment was reported to the web-based service clinical team who managed it according to the clinical incident policy.

During the clinical assessment, 154 participants reported no contraindications, and all except 2 reported the same information at the evaluation assessment. The 2 exceptions were 2 women who were taking the contraceptive pill.

During the evaluation assessment, 45 individuals reported potential contraindications, and 31 of these omitted this from their clinical assessment (14 unreported medications, 6 allergies, 1 previous operation, and 10 genital symptoms). One of these contraindications, an allergy to peanuts, could have caused a serious interaction with the medication prescribed.

Of the 33 discrepancies between clinical and evaluation assessments, 15 (46%) occurred when the clinical assessment was completed via SMS text message, 8 (24%) via telephone, and 10 (30%) via the secure web form. Univariate analysis showed that method of clinical assessment, gender, and sexuality were independently associated with agreement between the clinical and evaluation assessment. When combined in a multivariable model, method of initial assessment, gender, and sexuality remained statistically significantly associated with agreement between the 2 assessments.

The adjusted odds of agreement between assessments were 78% less among participants who completed their clinical assessment via SMS text messaging compared with those who completed this via telephone. This result was statistically significant (see Table 2). We found no statistically significant difference between the odds of agreement between assessments completed via telephone and via the secure web form.

Of those who took their medication, 91.1% (175/192) correctly recalled advice to abstain from sex for 7 days after they and their partner had commenced treatment, 93.4% (184/197) recalled advice on partner notification, and 89.3% (176/197) had notified partners.

**Table 2:** Crude and adjusted odd ratios of agreement in reporting information.

Exposure variable		Crude odds ratio (95% CI)	*P* value	Adjusted odds ratio (95% CI)^a^	*P* value
**Method of initial assessment**					
	Call	1 (reference^b^)	⁠—^b^	1 (reference)	⁠—^b^
	SMS text message	0.29 (0.11-0.75)	.01	0.22 (0.08-0.61)	.004
	Secure web form	0.58 (0.21-1.57)	.28	0.50 (0.17-1.43)	.20
**Ever attended a clinic?**					
	Yes	1 (reference)	⁠—^b^	1 (reference)	⁠—^b^
	No	2.21 (0.81-6.07)	.12	—^d^	⁠—^d^
**Gender**					
	Female	1 (reference)	⁠—^b^	1 (reference)	⁠—^b^
	Male	2.66 (1.19-5.93)	.02	2.75 (1.16-6.56)	.02
**Age (years)**					
	18-19	1 (reference)	⁠—^b^	1 (reference)	⁠—^b^
	20-24	1.68 (0.52-5.40)	.38	—^d^	—^d^
	25-29	2.10 (0.58-7.55)	.26	—^d^	⁠—^d^
	30-34	1.31 (0.32-5.32)	.70	—^d^	⁠—^d^
	35+	1.25 (0.25-6.29)	.78	—^d^	⁠—^d^
**Ethnicity**					
	White	1 (reference)	⁠—^b^	1 (reference)	⁠—^b^
	Black British/Caribbean/African	0.87 (0.37-2.05)	.74	— ^d^	⁠—^d^
	Asian/Asian British	1.31 (0.15-11.66)	.81	—^d^	⁠—^d^
	Mixed/multiple groups	0.56 (0.19-1.71)	.31	—^d^	⁠—^d^
	Other/prefer not to say	3.19 (0.39-26.26)	.28	—^d^	⁠—^d^
**Sexuality**					
	Heterosexual	1 (reference)	⁠—^b^	1 (reference)	⁠—^b^
	Homosexual	0.69 (0.07-6.45)	.75	0.34 (0.03-3.65)	.37
	Bisexual	0.23 (0.07-0.72)	.01	0.19 (0.06-0.66)	.009
	Prefer not to say	—^c^	—^c^	—^c^	—^c^
**Index of multiple deprivation** **q** **uintile**					
	1 (most deprived)	1 (reference)	⁠—^b^	1 (reference)	⁠—^b^
	2	1.10 (0.47-2.56)	.83	—^d^	—^d^
	3	3.33 (0.70-15.90)	.13	—^d^	—^d^
	4	0.36 (0.10-1.28)	.13	—^d^	—^d^
	5 (least deprived)	—^c^	—^c^	—^d^	—^d^

^a^Only variables where crude odds ratios were found to be significant (*P*<.05) were carried into the multivariable model.

^b^Reference group.

^c^Not applicable, as variable was not carried into the multivariable model.

^d^Not applicable, as no sample participants within these categories.

### Time-From-Diagnosis-to-Treatment

From the 192 participants who took their treatment, median time-to-treatment was 4 days (IQR 3-5.5). A total of 91.7% (176/192) of those evaluated were treated within 7 days of receiving their positive diagnosis.

A Kruskal-Wallis H test was used to analyze the relationship between time-to-treatment and the medium of the clinical assessment as this variable was not normally distributed. Of the 192, 55 were assessed via SMS text message, 76 via telephone, and 61 via web form. There was a statistically significant difference between time-to-treatment by method of clinical assessment (*H_2_*=24.169; *P*<.001), with a median time-to-treatment of 2.5 days for SMS text messaging, 4 days for web form, and 4.5 days for telephone call. Similarly, statistically significant differences were found between SMS text message and telephone call (*H_2_*=21.990; *P*<.001) and SMS text message and web form (*H_2_*=14.702; *P*<.001). There was no statistically significant difference in the median time-to-treatment between web form and telephone call.

### User Acceptability

The majority of users (185/197, 93.9%) were happy with the information provided on treatment. Almost all users reported that they could both understand (196/199, 99.0%) and were comfortable (196/198, 98.5.0%) with the SMS text messages they received from the service. 19.4% (38/196) contacted SH:24 for support, and 89.9% (178/198) said they would use the web-based service again.

## Discussion

### Principal Findings

This service evaluation documented an iterative process of testing 3 methods of clinical assessment before remote prescribing. It showed that users are significantly more likely to report accurate clinical histories during a telephone consultation than through a single SMS text message. We did not find a statistically significant difference between the accuracy of clinical histories reported via telephone and via the secure web form. It showed that treatment via a web-based sexual health service in general, and SMS text message assessment in particular, supports rapid treatment that is well within the standards set in current guidance; the National Chlamydia Screening Programme (NCSP) audit standard aims for 95% of patients to have a time-to-treatment of 6 weeks or less [20,21]. An NCSP audit of chlamydia treatment services carried out in 2017 [22] found that 92.0% of the sample met this standard, whereas within this service evaluation, 91.7% (176/192) of those evaluated were treated within 7 days of receiving their positive diagnosis. This is comparable with findings from Estcourt et al’s [23] exploratory studies into an electronic sexual health clinic system for management, prevention, and control of STIs, where results were delivered via a secure web portal and treatment was collected from a local pharmacy.

Our findings are important as treatment is increasingly part of routine web-based sexual health services. This is reflected both in the use of the web-based service evaluated here and quality standards and guidelines recently released by relevant professional bodies.

Since the start of the service in 2017, 5130 chlamydia treatments have been delivered via SH:24 by post in 14 different areas of the country. Postal treatment is highly acceptable to service users, with 80% of those offered treatment by post taking up this offer. More information about the uptake and number of orders can be found in Multimedia Appendix 2. The service now uses the secure web form to assess eligibility, and SMS text messaging or telephone are no longer used, except to gain more detail on contraindications that have been reported in the form.

Since the development of the service, the Faculty of Sexual and Reproductive Healthcare and the British Association for Sexual Health and HIV (BASHH) have published the first quality standards for online and remote providers of sexual and reproductive health care [24]. These standards not only reiterate the General Medical Council’s (GMC’s) recommendations on safety and remote prescribing [5] but also highlight the *safety nets* services should have to navigate and mitigate risks that may come with remote consultations [24]. The service evaluated here has developed a *2-way process* around the secure web form that is now used as the clinical assessment for eligibility in the web-based remote chlamydia treatment service where free textboxes within the web form allow users to share, for example, any medication they may be taking. Furthermore, as per the GMC guidelines, they now ask patients for their consent to share treatment information with their general practitioner [25].

Other changes in the service since this evaluation reflect updated clinical guidelines; during the evaluation users were offered SDA as first-line treatment; however, in response to BASHH recommendations [21], the first-line treatment offer for uncomplicated urogenital, pharyngeal, and rectal chlamydia infections is now Doxycycline 100 mg bd for 7 days.

### Interpretation

Higher risks associated with the SMS text message assessment provide important learning. New technologies such as cross- platform messaging services (eg, WhatsApp) are increasingly used by businesses for secure communication with their customers and strategies for assessment before medical prescribing may develop in this direction. We recommend further investigation into the risks and benefits of these approaches. The SMS text messaging strategy used here was limited to a single (rather long) message that included multiple questions and required a single answer. This is suboptimal and is a function of the limitations of SMS text messaging technology. It was not possible to break up the message into individual SMS text messages in case the connection was lost halfway through the interaction. SMS text messages remain undelivered when the connection breaks, unlike, for example, WhatsApp, where messages are delivered as soon as the connection is resumed. web forms are commonly used for history taking before prescriptions. This evaluation suggests that this is a promising approach, but our sample size was insufficient to provide significant results, and future research should explore their performance in comparison with telephone and face-to-face assessment.

Our analysis assumes that telephone assessment by an experienced clinician is safe as this is standard practice, but it also suggests that even this method is associated with inaccuracies. Even face-to-face consultations are associated with medication errors (usually prescribing errors) in hospitals varying between 2% and 14% [26]. It is likely that no method is consistently reliable in obtaining an accurate history. Situations where the clinical history can be checked against previous health records have obvious advantages, but this is not possible in sexual health where information traditionally is not linked to the rest of the medical record as a strategy to maintain confidentiality [27].

### Limitations

A small evaluation of a rapidly evolving service, this service evaluation did not calculate sample size when conceived. Participants were not allocated to a method of clinical assessment; designed to judge current care provided, the service evaluation followed an evolving service, where 3 iterations of the service design allowed for retrospective exploration of factors associated with agreement in safe prescribing information, including method of clinical assessment. Although, even with a small sample, we did find a difference in odds of agreement between SMS text messaging and telephone call, a robust, considered piece of research, with a calculated sample size, would be needed to fully explore the relationship between method of clinical assessment and agreement in safe prescribing information. As methods were tested consecutively, users were not randomly allocated to a particular method. Hence, those carrying out the follow-up assessment may not have been blinded; a possible source of bias.

We recognize the role that reporting bias may have played in user evaluation assessments. Users asked to reflect on their symptoms, once they have been given a diagnosis, may recognize symptoms they previously may have been deemed unrelated. Several women reported abdominal pain in the evaluation assessment, that they had considered period pain before they received their positive diagnosis. Preexisting literature highlights poor knowledge of nature and symptoms of chlamydia infection [28-30]. In a study of 18- to 24-year olds, a third of respondents were unaware of the asymptomatic nature of chlamydia infection, and 80.2% of respondents failed to recognize lower abdominal pain as a potential symptom in women [30].

Although most participants of this service evaluation stated a preference for a remote treatment service, as the sample consists solely of those who self-identify as asymptomatic, this should not be generalized to everyone seeking treatment. Sexual health clinics remain an integral element of STI treatment and care, and web-based services should be well integrated with face-to-face services [31,32]. However, considering that at least 70% of women and 50% of men infected with chlamydia are asymptomatic [33,34], this shows acceptability in a large proportion of those who need treatment.

### Conclusions and Recommendations

Postal treatment is an acceptable and rapid treatment option for uncomplicated genital chlamydia. Clinical assessment via SMS text message before remote prescription may not be accurate or sufficient. As health care is delivered, strategies that support safe remote prescribing are increasingly important, as is their evaluation, which should be robust and carefully considered.

## Appendix

Multimedia Appendix 1 Service evaluation questions.

Multimedia Appendix 2 Information on chlamydia treatment service provision.

## Abbreviations

BASHH: British Association for Sexual Health and HIV
GMC: General Medical Council
NCSP: National Chlamydia Screening Programme
NHS: National Health Service
SDA: single dose of Azithromycin
SH: 24: Sexual Health 24 hours a day
STI: sexually transmitted infection
