# Supplementary material for: Communication Strategies Used to Obtain Clinical Histories Before Remotely Prescribing Antibiotics for Postal Treatment of Uncomplicated Genital Chlamydia: Service Evaluation
Source: J Med Internet Res. 2020 Jun 17;22(6):e15970. doi: 10.2196/15970 (PMC7330733; doi:10.2196/15970)
Supplement: Multimedia Appendix 1 [file jmir_v22i6e15970_app1.docx]

*Service evaluation questions*

1. Firstly, can I confirm your name and date of birth?

2. Did you receive the medication from SH:24 that you requested?

Yes/No

3. When did you receive the medication from SH:24?

Date and time

4. Did you take the medication that you received from SH:24?

Yes/No

5. When did you take the medication that you received from SH:24?

Date and time – calculate hours from time received until time taken – calculate hours between notification of result and treatment

6. Did you experience any side effects from taking this medication?

Yes/No, if Yes, free text description of side effects

7. Did you have any other problems in relation to taking this medication?

Yes/No, if Yes, free text description of side effects

8. Are you taking medicine or allergic to any medicine, soya or peanuts?

Yes/No

9. Do you have liver, kidney or heart problems or myasthenia gravis?

Yes/No

10. Are you pregnant or breastfeeding?

Yes/No

11. Do you have any symptoms: fever, joint, pelvic (lower abdominal) or anal pain?

Yes/No

12. When you received the text messages offering treatment were there any elements of these messages that were unclear or difficult to understand?

Yes/No, If Yes, free text

13. When you received the text messages offering treatment were there any questions that you did not feel confident to answer?

14. How much information was provided about your treatment

too much/too little/about right

15. Did you contact SH:24 during the process of ordering/receiving or taking the medication for further information/support?

Yes/No, If Yes, free text description of support + check clinical record

16. If you needed treatment for chlamydia in the future would you prefer to access your treatment online or during a clinic consultation?

Online/Clinic

17. Did you receive advice on abstaining from sex after the treatment?

Yes/No

18. For how long were you advised to abstain from sex?

Number of days

19. Did you receive advice on notifying partners?

Yes/No

20. What action have you taken to notify partners?

Did they go to SXT? Yes/No

21. Do you have any questions about the process of ordering or taking this medication?

Free text response
