# Supplementary material for: Communication Strategies Used to Obtain Clinical Histories Before Remotely Prescribing Antibiotics for Postal Treatment of Uncomplicated Genital Chlamydia: Service Evaluation
Source: J Med Internet Res. 2020 Jun 17;22(6):e15970. doi: 10.2196/15970 (PMC7330733; doi:10.2196/15970)
Supplement: Multimedia Appendix 2 [file jmir_v22i6e15970_app2.docx]

*Information on chlamydia treatment service provision*

| *Time period* | *Positive CT diagnoses and offers of postal treatment during period* | *Users who were eligible and chose postal treatment during period* | *Percentage uptake of postal treatment from eligible population* | *Number of areas of the UK where SH:24 delivers chlamydia treatments* |
| --- | --- | --- | --- | --- |
| Audit period  (15/02/17 and 24/10/17) | 581 | 321 | 55.2% | 2 |
| First year of service  (February 2017- end of January 2018) | 1,209 | 818 | 67.7% | 4 |
| Second year of service  (February 2018-  End of January 2019) | 3,034 | 2,463 | 81.2% | 9 |
